# Brain-spine interface for movement restoration after spinal cord injury

**DOI:** 10.1016/j.bas.2024.102926

**Published:** 2024-08-10

**Authors:** Thangavel Lakshmipriya, Subash C.B. Gopinath

**Affiliations:** Center for Global Health Research, Saveetha Medical College & Hospital, Saveetha Institute of Medical and Technical Sciences (SIMATS), Thandalam, Chennai, 602 105, Tamil Nadu, India; Center for Global Health Research, Saveetha Medical College & Hospital, Saveetha Institute of Medical and Technical Sciences (SIMATS), Thandalam, Chennai, 602 105, Tamil Nadu, India; Faculty of Chemical Engineering & Technology, Universiti Malaysia Perlis (UniMAP), Arau, 02600, Perlis, Malaysia; Institute of Nano Electronic Engineering, Universiti Malaysia Perlis (UniMAP), Kangar, 01000, Perlis, Malaysia; Department of Technical Sciences, Western Caspian University, Baku, AZ 1075, Azerbaijan

**Keywords:** Trauma, Brain disorder, Neurological disease

Dear Editor

Spinal cord injury (SCI) involves damage to the spinal cord caused by trauma or disease. It also causes nerve damage in the spinal cord and leads to cauda equina (Zhang et al., 2023). Since the spinal cord receives and sends signals between the body and the brain, SCI often causes permanent changes in sensation, strength, and other bodily functions. In particular, SCI disrupts communication between the brain and the spinal cord, leading to difficulties in walking and paralysis (Capogrosso et al., 2016). Various research efforts are ongoing to develop treatment options for repairing the spinal cord after injury. In the meantime, rehabilitation helps people with injuries lead independent and productive lives. In this context, brain-spine interfaces are a developing technology that helps patients walk naturally after SCI.

Brain-spine interfaces are a technology that uses epidural electrical stimulation (EES) to create a link between the spinal cord and the brain by transforming motor goals into muscle activation stimuli (Insausti-Delgado et al., 2022). The implanted electrodes are fixed on the outer area of the spinal cord. EES can release electrical inputs, making it possible to bypass disconnected and damaged pathways by indirectly transmitting sensory signals to the motor pools through the proprioceptive fibers and altering the excitability of the spinal cord. Brain-spine interfaces use brain activity to control spinal stimulation at the injury site, mimicking the command transfer from the brain to the spinal cord. The signals for motor intentions, released from the brain, are captured by the brain-spine interfaces and translated into commands for spinal stimulation. In brain-spine interface technology, the command originates in the brain, where signals linked to motor intent are identified by brain-computer interfaces. The signals are further decoded into commands with movement patterns and then transmitted to an implanted electrode along the spinal cord. This electrode activates the spinal circuits by generating electrical pulses, helping to initiate movement based on the individual's intent. This system enables natural control over leg movements to walk, stand, and climb stairs.

Brain-face interfaces provide a new life for severely paralyzed patients. Traditional treatments are unable to improve or repair the neural pathways. Brain-spine connections reduce neurological deficits and help to repair damaged sensorimotor pathways. Researchers are improving the system of brain-spine interface technology for utilization in spinal cord injury recoveries. Several studies have focused on and developed brain-face interfaces, proving them to be an effective way of improving patient movement control. Flint et al. developed a brain-face interface and applied it in two monkeys using a local field potentials (LFPs) decoder to create accurate and stable control over 350 days (Flint et al., 2013). In another study, an effective brain-face interface technology for stroke rehabilitation was developed based on Hebbian principles of associativity. The common peroneal nerve was stimulated with a magnetic stimulator with the cathode proximal. Surface electrodes were utilized to record the activity of the electromyographic tibialis anterior muscle. This system helps improve functional plasticity and motor function after stroke (Mrachacz-Kersting et al., 2016). At present, brain-face interfaces are transitioning from theoretical to practical experiments. Clinical trials with humans affected by SCI have shown promising outcomes. For instance, Lorach et al. developed a wireless digital bridge between the spinal cord and brain, which allows volitional control over the amplitude and timing of muscle activity, providing adaptive and natural control over walking and standing activity for patients with spinal cord injury (Lorach et al., 2023). This research involved a patient affected by a traumatic cervical spine injury from a cycling accident. They used a brain-spine interface comprised of two systems, namely WIMAGINE and ACTIVA. WIMAGINE is a brain system consisting of two internal implants fixed on each side of the brain, with two external antennas attached to the implants. The antennas and implants recognize and transfer electrical pulses from the brain to ACTIVA, the digital bridge fixed in the spine. A machine learning model was trained to identify the brain activity linked to leg control, and this activity was controlled with the electrodes implanted into the brain. The electrodes record the cortical activity and stimulate the lumbosacral spinal cord, allowing the patient to move independently. With this system, the participant regained the ability to walk with the support of crutches even when the brain-spine interfaces were switched off. Expanding the concept of a digital bridge to the cervical spinal cord may make it possible to restore arm and hand movements after a stroke or spinal cord injury, which holds promise for enhancing the quality of life for those affected by spinal cord injuries.

## Declaration of competing interest

The authors declare that they have no known competing financial interests or personal relationships that could have appeared to influence the work reported in this paper.
